# Pathologic assessment of tumor-associated macrophages and their histologic localization in invasive breast carcinoma

**DOI:** 10.1186/s43046-020-0018-8

**Published:** 2020-01-27

**Authors:** Shorouk E. Mwafy, Dina M. El-Guindy

**Affiliations:** Pathology Department, Faculty of Medicine, Tanta University, Tanta, Egypt

**Keywords:** Tumor-associated macrophages (TAMs), CD163, CD68, Breast cancer, Histologic localization

## Abstract

**Background:**

Tumor-associated macrophages (TAMs) are important in regulating cross-talk between tumor cells and tumor microenvironment. TAMs are involved in multiple steps of tumor progression and invasion. This study aimed to compare CD163 expression with the widely used CD68 pan-macrophage marker in invasive breast carcinoma. Furthermore, it focused on assessing the significance of TAMs localization in relation to clinicopathological parameters.

**Results:**

CD68 and CD163 immunohistochemical expressions within TAMs infiltrating both tumor nest (TN) and tumor stroma (TS) were evaluated in 60 specimens with invasive breast carcinoma. High CD68-positive stromal TAMs was significantly related to larger tumor, nodal metastasis and vascular invasion (*p* = 0.003, 0.037, 0.032, respectively), whereas high CD163-positive stromal TAMs was significantly related to larger tumors, nodal metastasis, stage III tumors, vascular invasion, estrogen receptor (ER) negativity, and triple-negative subtype (*p* = 0.023, < 0.001, 0.001, 0.022, 0.002, 0.017, respectively). On multivariate analysis, high CD68-positive TAMs infiltrating TS was significantly associated with larger tumor and positive nodal metastasis (*p* = 0.006 and 0.016, respectively), whereas high CD163 TAMs density within TS was significantly associated with positive vascular invasion, nodal metastasis, and molecular subtypes (*p* = 0.003, 0.001, and 0.009, respectively).

**Conclusion:**

TAMs within tumor stroma and tumor nest have different levels of association with poor prognostic parameters. So, it is of great importance to consider the histologic localization of TAMs in addition to the degree of TAMs infiltration.

## Background

Breast cancer (BC) is the most commonly diagnosed cancer and the main cause of cancer-related deaths in females worldwide [1]. Initial studies investigating mechanisms responsible for BC metastatic potential and treatment resistance have focused attention on tumor cells themselves. However, the role of the tumor microenvironment (TME) in tumor progression and treatment resistance have been recently identified [2].

Tumors are composed of malignant and non-malignant cells, which constitute the tumor microenvironment [3]. Tumor-associated macrophages (TAMs) are crucial regulators of cancer cells and microenvironment. They are highly plastic cells that are affected by and are reprogrammed by signals found within TME. Chemotactic factors attract TAMs to tumors which are a rich source of cytokines and proteases for the promotion of invasion, tumor angiogenesis, immune evasion, and suppression of apoptosis [4]

TAMs have a potential ability to differentiate into either M1- or M2-polarized macrophages, which have opposing effects on tumor progression. Classically, activated M1 macrophages release pro-inflammatory cytokines and activate type1 T cell response that has a cytotoxic effect on tumor cells, whereas M2 macrophages produce proteolytic enzymes, suppress immune response, and contribute to hypoxia-induced angiogenesis, thus promoting tumor cell proliferation and migration [5].

CD68 and CD163 are glycoproteins that are expressed in human monocytes and tissue macrophages. CD68 is a pan-macrophage marker that recognizes both M1 and M2 macrophages, while CD163 is a highly specific monocyte/macrophage marker for polarized M2 macrophages [6, 7].

Previous studies demonstrated that TAMs infiltration is associated with poor clinical outcomes in breast cancer. Also, high level of TAMs infiltration was associated with negative hormone receptor status [8]. TAMs have been considered as a potential target for adjuvant therapy [5].

This study aimed to evaluate CD163 expression compared to the widely used CD68 pan-macrophage marker in invasive breast carcinoma. In addition, it focused on assessing the significance of TAMs localization in relation to clinicopathologic parameters.

## Methods

This retrospective study included 60 primary BC specimens. Formalin-fixed paraffin-embedded blocks were retrieved from the Pathology Department during the period from January 2018 to June 2019. Approval of the institutional ethics committee was obtained.

Eligibility in this study included patients who had pathologically confirmed invasive breast carcinoma of no special type, proper histologic specimens with sufficient tumor tissue, and complete clinicopathologic data. Patients who received chemotherapy or radiotherapy prior to surgery were excluded.

Clinicopathologic data of these patients were obtained from their medical reports. All included cases were classified as invasive carcinoma of no special type according to the World Health Organization (WHO) criteria [9]. Nottingham grading system was used to determine tumor grade [9, 10]***.*** TNM staging was assessed according to the American Joint Committee on Cancer [11]***.*** Molecular subtypes were categorized as luminal A, luminal B, HER2+, and triple-negative subtype according to the modern molecular classification [12]***.***

### Immunohistochemical staining

Sections (5 um thick) were prepared on positively charged slides and then left to dry for 30 min at 37 °C. Dako PT Link unit was applied for deparaffinization and antigen retrieval. Both high and low pH EnVisionTM FLEX Target Retrieval Solutions were used reaching 97 °C for 20 min. Immunostaining was carried out with Dako Autostainer Link 48. Antibodies included in this study were CD68 mouse monoclonal antibody (M0876, Dako, Glostrup, Denmark) and CD163 rabbit monoclonal antibody (clone EP324, Medaysis, CA, USA). In brief, slides were left in Peroxidase-Blocking Reagent for 5 min, incubated with primary antibodies for 20–30 min, horseradish peroxidase (HRP) polymer reagent for 20 min, and diaminobenzidine (DAB) chromogen/substrate working solution for 10 min. Finally, counterstaining with hematoxylin was done.

### Quantification of TAMs

All CD68 and CD163 stained slides were examined to determine areas with the highest levels of tumor-associated macrophages (TAMs) infiltration. For each case, three hotspots in a high-power field (× 400) were decided for counting TAMs. TAMs were counted manually using the plug-in “cell counter” in the ImageJ software. TAMs were counted in both tumor nest (TN) and tumor stroma (TS). TAMs within TN represent macrophages within tumor cell nests and indirect contact with tumor cells, whereas stromal TAMs were defined as macrophages infiltrating tumor stroma of the invasive carcinoma. Cases were then assigned into low and high groups considering the median density of TAMs infiltration in both TN and TS as a cut-off point [13].

Cases were further grouped according to CD68- and CD163-positive TAMs density in both TN and TS as follows: TN^Low^ and TS^Low^ included cases with low TAMs density in both TN and TS, TN^High^ and TS^Low^ included cases with high TAMs density in TN and low TAMs density in TS, TN^High^ and TS^High^ included cases with high TAMs density in both TN and TS, TN^Low^ and TS^High^ included cases with low TAMs density in TN and high TAMs density in TS.

### Statistical analysis

Statistical analysis was performed using Statistical Package for Social Science (SPSS version 23.0). Data were presented as mean ± SD for numerical variables and frequencies for categorical ones. Analyzing relations between TAMs infiltration and clinicopathologic variables was carried out using chi-square (*χ*^*2*^). Fisher exact and Monte-Carlo tests were used when appropriate. Column proportion test was used for pairwise comparisons when the omnibus test was significant. The *p* values were adjusted with Bonferroni method. Normality of numerical variables was determined using Shapiro-Wilk test. Independent student *t* test and analysis of variance (ANOVA) test were used to compare means of numerical variables. A multivariate logistic regression analysis was performed to identify the variables that were independently associated with TAMs infiltration. *p* values < 0.05 were considered statistically significant.

## Results

### Clinicopathologic characteristics

Clinicopathologic characteristics of the studied cases are illustrated in Table 1. Mean age of the studied BC cases was 51.22 + 11.64 years. In 39 (65%) cases, tumor measured < 5 cm in its greatest dimension. GII tumors constituted 32 (53.3%) cases whereas 28 (46.7%) cases were poorly differentiated. Concerning tumor stage, 25 (41.7%) cases were stage III, 32 (53.3%) cases were stage II, while only 3 (5%) cases were stage I. Positive nodal metastasis was detected in 34 (56.7%) cases, whereas positive vascular invasion was identified in 38 (63.3%) cases.

**Table 1:** Clinicopathological characteristics of the studied cases

Variable	Total
Age (years) mean ± SD	51.22 ± 11.64
Size	
<5 cm	39 (65)
≥5 cm	21 (35)
Grade	
GII	32 (53.3)
GIII	28 (46.7)
Nodal metastasis	
Negative	26 (43.3)
Positive	34 (56.7)
Staging	
I	3 (5)
II	32 (53.3)
III	25 (41.7)
Vascular invasion	
Negative	22 (36.7)
Positive	38 (63.3)
ER	
Negative	13 (21.7)
Positive	47 (78.3)
PR	
Negative	26 (43.3)
Positive	34 (56.7)
Her2	
Negative	45 (75)
Positive	15 (25)
Ki-67	
≤14%	29 (48.3)
>14%	31 (51.7)
Molecular subtypes	
Luminal A	27 (45)
Luminal B	24 (40)
Triple negative	9 (15)

*ER* estrogen receptor, *PR* progesterone receptor

As regards hormone receptor status, 47 (78.3%) cases were estrogen receptor (ER)-positive and 34 (56.7%) positively expressed progesterone receptor (PR). Forty-five (75%) cases were negative for Her2 expression. Ki-67 proliferation index was > 14% in 31 (51.7%) cases and ≤ 14% in the remaining 29 (48.3%) cases. Referring to the molecular subtypes, 27 (45%) cases were luminal A, 24 (40%) cases were luminal B while the remaining 9 (15%) cases were triple-negative tumors.

### Relation between CD68 expression in both tumor nest and stroma and clinicopathologic characteristics

**Fig. 1:**
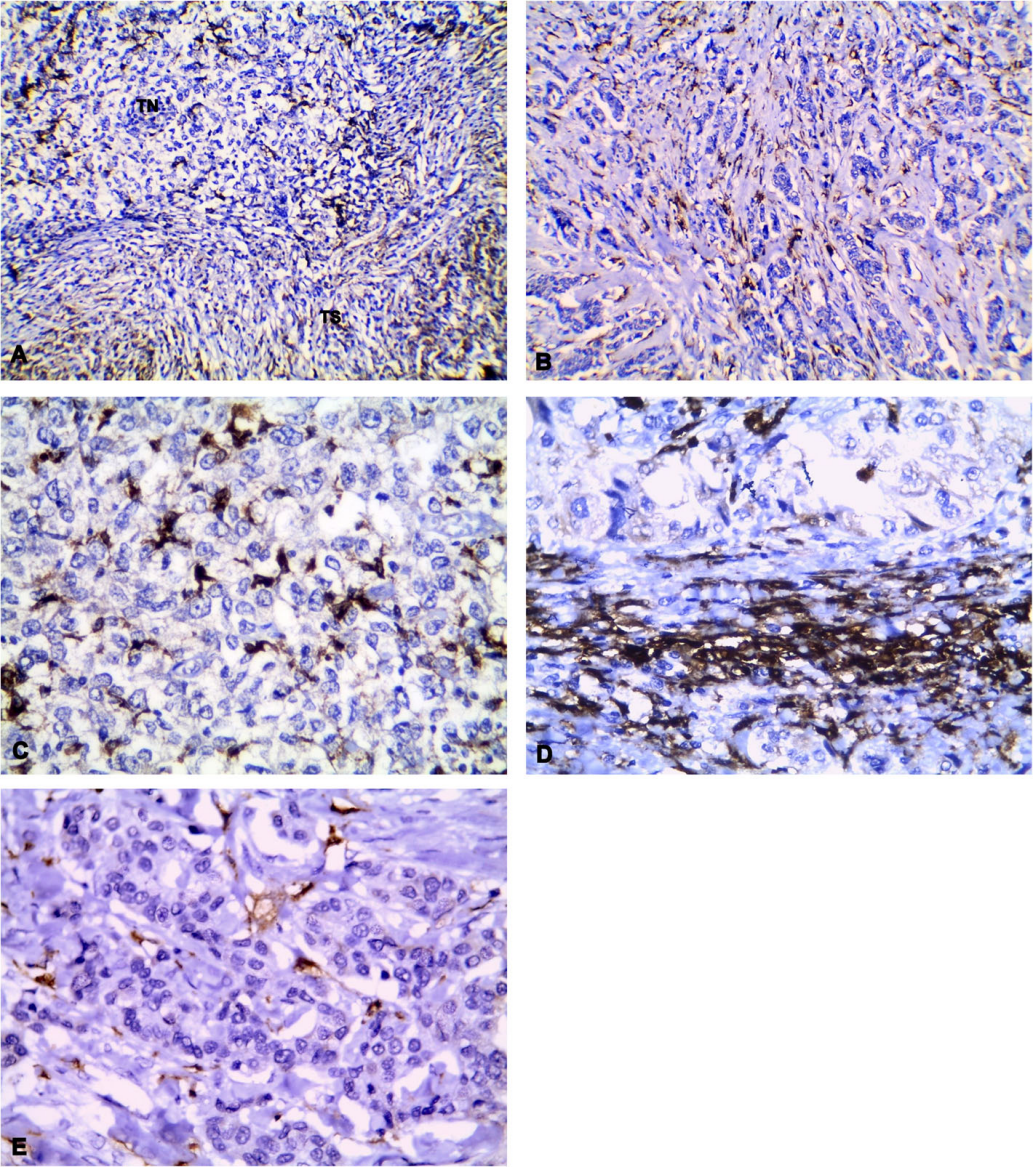
CD68-positive tumor-associated macrophages (TAMs). **a** CD68-positive TAMs mainly in tumor stroma (TS) with few within tumor nest (TN) in GIII case (× 200). **b** CD68-positive TAMs within tumor stroma in GII case (× 200). **c** High density of CD68-positive TAMs within tumor nest in GIII case (× 400). **d** High density of CD68-positive TAMs infiltrating tumor stroma in GIII case (× 400). **e** Low density of CD68-positive TAMs in both tumor nest and stroma in GII case (× 400)

Significant relations were detected between CD68-positive TAMs infiltration within TS and tumor size, nodal metastasis and vascular invasion. CD68-positive TAMs infiltrating TS was significantly higher in 16 (76.2%) tumors measuring > 5 cm in its greatest dimension (*p* = 0.003), 21 (61.8%) cases positive for nodal metastasis (*p* = 0.037) and 23 (60.5%) tumors with positive vascular invasion (*p* = 0.032). On the contrary, CD68-positive TAMs in TN was not significantly associated with any clinicopathological features. On multivariate logistic regression analysis, high CD68-positive stromal TAMs was significantly associated with larger tumor (OR = 0.044; 95% CI = 0.005–0.413; *p* = 0.006) and positive nodal metastasis (OR = 0.074; 95% CI = 0.009–0.621; *p* = 0.016) (Tables 2 and 3, Fig. 1).

**Table 2:** Relation between CD68 expression in both tumor nest and stroma and clinicopathologic characteristics

Variable	Total	CD68 in tumor nest			CD68 in tumor stroma		
		Low *N* = 31 *N* (%)	High *N* = 29 *N* (%)	*p*	Low *N* = 30 *N* (%)	High *N* = 30 *N* (%)	*p*
Age (years) mean ± SD		52.81 ± 11.88	49.52 ± 11.34	0.278	52.17 ± 12.19	50.27 ± 11.19	0.532
Size							
< 5 cm	39	23 (59)	16 (41)	0.123	25 (64.1)	14 (35.9)	0.003*
≥ 5 cm	21	8 (38.1)	13 (61.9)		5 (23.8)	16 (76.2)	
Grade							
GII	32	18 (56.3)	14 (43.8)	0.448	18 (56.3)	14 (43.8)	0.301
GIII	28	13 (46.4)	15 (53.6)		12 (42.9)	16 (57.1)	
Nodal metastasis							
Negative	26	17 (65.4)	9 (34.6)	0.063	17 (65.4)	9 (34.6)	0.037*
Positive	34	14 (41.2)	20 (58.8)		13 (38.2)	21 (61.8)	
Staging							
I	3	2 (66.7)	1 (33.3)	0.118	3 (100)	0 (0)	0.153
II	32	20 (62.5)	12 (37.5)		17 (53.1)	15 (46.9)	
III	25	9 (36)	16 (64)		10 (40)	15 (60)	
Vascular invasion							
Negative	22	9 (40.9)	13 (59.1)	0.205	15 (68.2)	7 (31.8)	0.032*
Positive	38	22 (57.9)	16 (42.1)		15 (39.5)	23 (60.5)	
ER							
Negative	13	6 (46.2)	7 (53.8)	0.653	7 (53.8)	6 (46.2)	0.754
Positive	47	25 (53.2)	22 (46.8)		23 (48.9)	24 (51.1)	
PR							
Negative	26	14 (53.8)	12 (46.2)	0.768	14 (53.8)	12 (46.2)	0.602
Positive	34	17 (50)	17 (50)		16 (47.1)	18 (52.9)	
Her2							
Negative	45	22 (48.9)	23 (51.1)	0.456	23 (51.1)	22 (48.9)	0.766
Positive	15	9 (60)	6 (40)		7 (46.7)	8 (53.3)	
Ki-67							
≤ 14%	29	16 (55.2)	13 (44.8)	0.599	15 (51.7)	14 (48.3)	0.796
> 14%	31	15 (48.4)	16 (51.6)		15 (48.4)	16 (51.6)	
Molecular subtypes							
Luminal A	27	15 (55.6)	12 (44.4)	0.535	14 (51.9)	13 (48.1)	0.475
Luminal B	24	13 (54.2)	11 (45.8)		10 (41.7)	14 (58.3)	
Triple negative	9	3 (33.3)	6 (66.7)		6 (66.7)	3 (33.3)	

*ER* estrogen receptor, *PR* progesterone receptors

*Statistically significant

**Table 3:** Multivariate analysis of clinicopathological data and CD68 positive TAMs within tumor stroma

Variables	OR (95% CI)	*p*
Size	0.044 (0.005–0.413)	0.006*
Nodal metastasis	0.074 (0.009–0.621)	0.016*
Vascular invasion	1.364 (0.241–7.734)	0.726

*CI* confidence interval, *OR* odds ratio, *TAMs* tumor-associated macrophage

*Statistically significant

### Relation between combined densities of CD68-positive TAMs in both tumor nest and stroma and clinicopathologic characteristics

Tumors with a combined high density of CD68-positive TAMs in both TN and TS were significantly associated with larger tumors (*p* = 0.001). In pairwise comparison, CD68-positive TN^High^ and TS^High^ cases showed the highest proportion of tumors measuring ≥ 5 cm in their greatest dimension (57.2%) compared to cases with CD68-positive TN^Low^ and TS^Low^ (19%), TN^High^ and TS^Low^ (4.8%), and TN^Low^ and TS^High^ (19%) (pairwise *p* = 0.003, < 0.001, 0.026, respectively). As regards vascular invasion, CD68-positive TN^High^ and TS^High^ cases were significantly higher in cases with positive vascular invasion compared to CD68-positive TN^High^ and TS^Low^ ones (pairwise *p* = 0.048). These relations are demonstrated in Table 4.

**Table 4:** Relation between combined densities of CD68 expression in both tumor nest and stroma and clinicopathologic characteristics

	Total	CD68-positive TAMs				*p*
		TN^Low^ and TS^Low^ A *N* = 17 *N* (%)	TN^High^ and TS^Low^ B *N* = 13 *N* (%)	TN^High^ and TS^High^ C *N* = 16 *N* (%)	TN^Low^ and TS^High^ D *N* = 14 *N* (%)	
Age (years) mean ± SD		52.88 ± 13.71	51.23 ± 10.32	48.13 ± 12.25	51.22 ± 11.64	0.646
Size						
< 5 cm	39	13 (33.3)	12 (30.8)	4 (10.3)	10 (25.6)	0.001*
≥ 5 cm	21	4 (19)	1 (4.8)	12 (57.2)	4 (19)	
Pairwise comparison: AB: 0.248, AC: 0.003*, AD:0.749, BC: < 0.001*, BD: 0.326, CD: 0.026*						
Grade						
GII	32	10 (31.3)	8 (25)	6 (18.7)	8 (25)	0.545
GIII	28	7 (25)	5 (17.9)	10 (35.7)	6 (21.4)	
Nodal metastasis						
Negative	26	12 (46.2)	5 (19.2)	4 (15.4)	5 (19.2)	0.053
Positive	34	5 (14.7)	8 (23.5)	12 (35.3)	9 (26.5)	
Stage						
I	3	2 (66.7)	1 (33.3)	0	0	0.119
II	32	10 (31.3)	7 (21.9)	5 (15.5)	10 (31.3)	
III	25	5 (20)	5 (20)	11 (44)	14 (16)	
Vascular invasion						
Negative	22	6 (27.3)	9 (40.9)	4 (18.2)	3 (13.6)	0.048*
Positive	38	11 (28.9)	4 (10.5)	12 (31.7)	11 (28.9)	
Pairwise comparison: AB: 0.139, AC: 0.707, AD: 0.456, BC: 0.027*, BD: 0.021*, CD:1						
ER						
Negative	13	3 (23.1)	4 (30.7)	3 (23.1)	3 (23.1)	0.862
Positive	47	14 (29.8)	9 (19.1)	13 (27.7)	11 (23.4)	
PR						
Negative	26	7 (26.9)	7 (26.9)	5 (19.3)	7 (26.9)	0.628
Positive	34	10 (29.4)	6 (17.6)	11 (32.4)	14 (20.6)	
Her2						
Negative	45	12 (26.7)	11 (24.4)	12 (26.7)	10 (22.2)	0.845
Positive	15	5 (33.3)	2 (13.3)	4 (26.7)	4 (26.7)	
Ki-67						
≤ 14%	29	8 (27.6)	7 (24.1)	6 (20.7)	8 (27.6)	0.745
> 14%	31	9 (29)	6 (19.4)	10 (32.2)	6 (19.4)	
Molecular subtypes						
Luminal A	27	7 (25.9)	7 (25.9)	5 (18.6)	8 (29.6)	0.284
Luminal B	24	8 (33.3)	2 (8.4)	9 (37.5)	5 (20.8)	
Triple negative	9	2 (22.2)	4 (44.5)	2 (22.2)	1 (11.1)	

*ER* estrogen receptor, *PR* progesterone receptor, *TAMs* tumor-associated macrophage, *TN* tumor nest, *TS* tumor stroma

*Statistically significant

### Relation between CD163 expression in both tumor nest and stroma and clinicopathologic characteristics

**Fig. 2:**
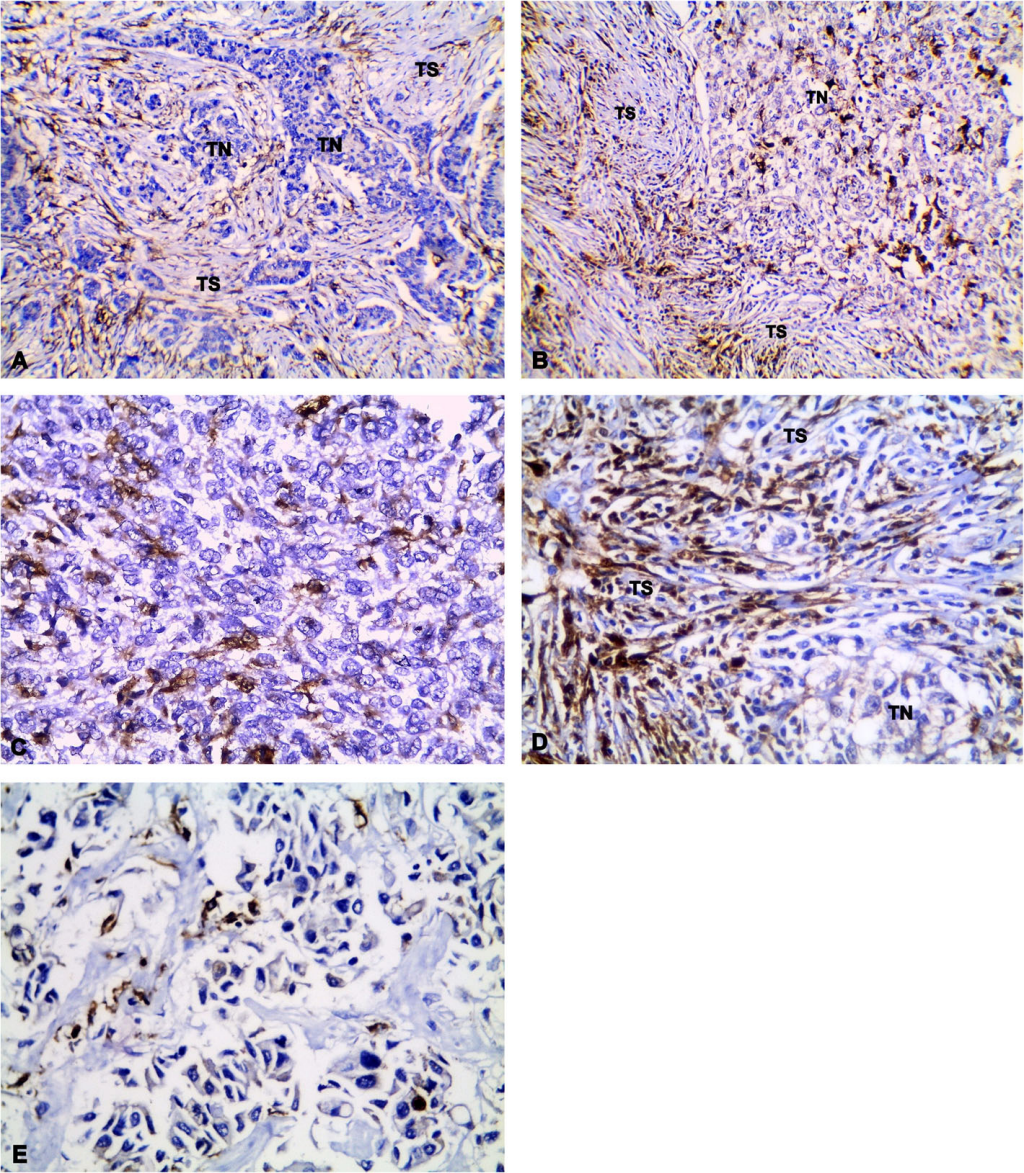
CD163-positive tumor-associated macrophages (TAMs). **a** CD163-positive TAMs mainly in tumor stroma (TS) with few within tumor nest (TN) in GII case (× 200). **b** CD163-positive TAMs within both tumor nest and stroma in GIII case (× 200). **c** High density of CD163-positive TAMs infiltrating tumor nest in GIII case (× 400). **d** High density of CD163-positive TAMs within tumor stroma in GIII tumor (× 400). **e** Low density of CD163-positive TAMs in both tumor nest and stroma in GII case (× 400)

There were statistically significant associations between CD163-positive TAMs infiltrating TS and tumor size, lymph node (LN) metastasis, tumor stage, and vascular invasion. High density of CD163-positive TAMs infiltrating TS was detected in 14 (66.7%) tumors measuring ≥ 5 cm in their greatest dimension (*p* = 0.023), 23 (67.6%) cases with positive LN metastasis (*p* < 0.001), 18 (72%) stage III cases (*p* = 0.001), and in 22 (57.9%) cases in which vascular invasion was identified (*p* = 0.022). Moreover, 11 (84.6%) ER-negative cases displayed high density of CD163-positive TAMs in TS (*p* = 0.002). As regards molecular subtypes, CD163-positive TAMs in TS was significantly high in triple-negative cases and low in luminal A cases (*p* = 0.017). Dealing with CD163-positive TAMs within TN, high density was detected in 19 (67.9%) grade III tumors (*p* = 0.005), and in 21 (67.7%) tumors with high ki-67 proliferation index (*p* = 0.002). Also, a significant association was observed between low density of CD163-positive TAMs within TN and luminal A subtype (*p* = 0.001). On multivariate logistic regression analysis, high CD163 TAMs density within TS was significantly associated with positive vascular invasion (OR = 0.026; 95% CI = 0.002–0.284; *p* = 0.003), nodal metastasis (OR = 0.068; 95% CI = 0.014–0.331; *p* = 0.001), and molecular subtypes (*p* = 0.009), whereas no significant associations were detected between CD163-positive TAMs within TN and clinicopathologic characteristics (Tables 5 and 6, Fig. 2).

**Table 5:** Relation between CD163 expression in both tumor nest and stroma and clinicopathologic characteristics

Variable	Total	CD163 in tumor nest			CD163 in tumor stroma		
		Low *N* = 31 *N* (%)	High *N* = 29 *N* (%)	*p*	Low *N* = 32 *N* (%)	High *N* = 28 *N* (%)	*p*
Age (years) mean ± SD		52.68 ± 12.99	49.66 ± 9.99	0.319	49.41 ± 11.92	53.29 ± 11.15	0.200
Size							
< 5 cm	39	21 (53.8)	18 (46.2)	0.645	25 (64.1)	14 (35.9)	0.023*
≥ 5 cm	21	10 (47.6)	11 (52.4)		7 (33.3)	14 (66.7)	
Grade							
GII	32	22 (68.8)	10 (31.3)	0.005*	18 (56.3)	14 (43.7)	0.628
GIII	28	9 (32.1)	19 (67.9)		14 (50)	14 (50)	
Nodal metastasis							
Negative	26	15 (57.7)	11 (42.3)	0.414	21 (80.8)	5 (19.2)	<0.001*
Positive	34	16 (47.1)	18 (52.9)		11 (32.4)	23 (67.6)	
Staging							
I	3	3 (100)	0 (0)	0.324	3 (100)	0 (0)	0.001*
II	32	16 (50)	16 (50)		22 (68.8)	10 (31.2)	
III	25	12 (48)	13 (52)		7 (28)	18 (72)	
Vascular invasion							
Negative	22	13 (59.1)	9 (40.9)	0.381	16 (72.7)	6 (27.3)	0.022*
Positive	38	18 (47.4)	20 (52.6)		16 (42.1)	22 (57.9)	
ER							
Negative	13	5 (38.5)	8 (61.5)	0.282	2 (15.4)	11 (84.6)	0.002*
Positive	47	26 (55.3)	21 (44.7)		30 (63.8)	17 (36.2)	
PR							
Negative	26	13 (50)	13 (50)	0.821	12 (46.2)	14 (53.8)	0.330
Positive	34	18 (52.9)	16 (47.1)		20 (58.8)	14 (41.2)	
Her2							
Negative	41	25 (55.6)	20 (44.4)	0.296	21 (46.7)	24 (53.3)	0.084
Positive	19	6 (40)	9 (60)		11 (73.3)	4 (26.7)	
Ki-67							
≤ 14%	29	21 (72.4)	8 (27.6)	0.002*	17 (58.6)	12 (41.4)	0.427
> 14%	31	10 (32.3)	21 (67.7)		15 (48.4)	16 (51.6)	
Molecular subtypes							
Luminal A	27	21 (77.8)	6 (22.2)	0.001*	15 (55.6)	12 (44.4)	0.017*
Luminal B	24	6 (25)	18 (75)		16 (66.7)	8 (33.3)	
Triple negative	9	4 (44.4)	4 (55.6)		1 (11.1)	8 (88.9)	

*ER* estrogen receptor, PR progesterone receptor

*Statistically significant

**Table 6:** Multivariate analysis of clinicopathological data and CD163 positive TAMs within tumor stroma

Variables	OR (95% CI)	*p*
Step 1		
Nodal metastasis	0.114 (0.034–0.382)	<0.001*
Step 2		
Nodal metastasis	0.121 (0.033–0.448)	0.002*
ER	8.859 (1.492–52.287)	0.016*
Step 3		
Vascular invasion	0.085 (0.014–0.495)	0.006*
Nodal metastasis	0.091 (0.021–0.389)	0.001*
ER	25.52 (2.528–257.5)	0.006*
Step 4		
Vascular invasion	0.028 (0.003–0.315)	0.004*
Nodal metastasis	0.071 (0.015–0.348)	0.001*
Molecular subtypes		0.082
Luminal A	0.026 (0.001–2.381)	0.113
Luminal B	0.009 (0.001–0.818)	0.041*
Triple negative	Reference	
ER	2.966 (0.147–59.96)	0.478
Step 5		
Vascular invasion	0.026 (0.002–0.284)	0.003*
Nodal metastasis	0.068 (0.014–0.331)	0.001*
Molecular subtypes		0.009*
Luminal A	0.008 (0.001–0.231)	0.005*
Luminal B	0.003 (0.001–0.124)	0.002*
Triple negative	Reference	

*CI* confidence interval, *ER* estrogen receptor, *OR* odds ratio, *TAMs* tumor-associated macrophage

*Statistically significant

### Relation between combined densities of CD163-positive TAMs in both tumor nest and stroma and clinicopathologic characteristics

CD163-positive TN^High^ and TS^High^ cases were significantly associated with GIII tumors, positive nodal metastasis, stage III tumors, ER negativity, high Ki-67 proliferation index compared to CD163-positive TN^Low^ and TS^Low^ (pairwise *p* = 0.029, 0.003, 0.018, 0.011, 0.012, respectively) denoting that high density of CD163-positive is associated with poor prognostic parameters. CD163-positive TN^High^ and TS^High^ cases were associated with higher GIII tumors compared to CD163-positive TN^Low^ and TS^High^ (pairwise *p* = 0.007). Also, CD163-positive TN^Low^ and TS^High^ cases were significantly related to positive nodal metastasis and stage III tumors compared to CD163-positive TN^Low^ and TS^Low^ (pairwise *p* = 0.011 and 0.018, respectively). CD163-positive TN^High^ and TS^Low^ cases were significantly associated with high Ki-67 compared to CD163-positive TN^Low^ and TS^Low^ (pairwise *p* = 0.012). Regarding molecular subtypes, CD163-positive TN^Low^ and TS^Low^ had the highest proportion of luminal A tumors (48.2%) compared to CD163-positive TN^High^ and TS^Low^ (7.4%) and TN^High^ and TS^High^ cases (14.8%) (pairwise *p* = 0.002 and 0.011, respectively), whereas CD163-positive TN^Low^ and TS^High^ cases had the highest proportion of triple-negative cases (44.4%) compared to CD163-positive TN^High^ and TS^Low^ (11.2%) (pairwise *p* = 0.002) as shown in Table 7.

**Table 7:** Relation between combined densities of CD163 expression in both tumor nest and stroma and clinicopathologic characteristics

	Total	CD163-positive TAMs				*p*
		TN^Low^ and TS^Low^ A *N* = 17 *N* (%)	TN^High^ and TS^Low^ B *N* = 15 *N* (%)	TN^High^ and TS^High^ C *N* = 14 *N* (%)	TN^Low^ and TS^High^ D *N* = 14 *N* (%)	
Age (years) mean ± SD		52.06 ± 11.53	46.00 ± 11.82	53.57 ± 5.71	53.43 ± 14.98	0.245
Size						
< 5 cm	39	14 (35.9)	11 (28.3)	7 (17.9)	7 (17.9)	0.136
≥ 5 cm	21	3 (14.4)	4 (19)	7 (33.3)	7 (33.3)	
Grade						
GII	32	11 (34.4)	7 (21.9)	3 (9.3)	11 (34.4)	0.014*
GIII	28	6 (21.4)	8 (28.6)	11 (39.3)	3 (10.7)	
Pairwise comparison: AB:0.476, AC:0.029*, AD: 0.456, BC:0.245, BD: 0.128, CD: 0.007*						
Nodal metastasis						
Negative	26	12 (46.2)	9 (34.6)	2 (7.7)	3 (11.5)	0.002*
Positive	34	5 (14.7)	6 (17.6)	12 (35.3)	11 (32.4)	
Pairwise comparison: AB: 0.712, AC:0.003*, AD:0.011*, BC: 0.021*, BD: 0.60, CD: 1						
Stage						
I	3	3 (100)	0 (0)	(0)	(0)	0.010*
II	32	11 (34.4)	11 (34.4)	5 (15.6)	5 (15.6)	
III	25	3 (12)	4 (16)	9 (36)	9 (36)	
Pairwise comparison: AB: 0.219, AC: 0.018*, AD: 0.018*, BC:0.126, BD: 0.126, CD: 1						
Vascular invasion						
Negative	22	10 (45.5)	6 (27.3)	3 (13.6)	3 (13.6)	0.097
Positive	38	7 (18.5)	9 (23.7)	11 (28.9)	11 (28.9)	
ER						
Negative	13	1 (7.7)	1 (7.7)	7 (53.8)	4 (30.8)	0.012*
Positive	47	16 (34)	14 (29.8)	7 (14.9)	10 (21.3)	
Pairwise comparison: AB: 1, AC: 0.011*, AD: 0.148, BC: 0.014*, BD:0.169, CD: 0.441						
PR						
Negative	26	6 (23.1)	6 (23.1)	7 (26.9)	7 (26.9)	0.779
Positive	34	11 (32.4)	9 (26.4)	7 (20.6)	7 (20.6)	
Her2						
Negative	45	13 (28.8)	8 (17.8)	12 (26.7)	12 (26.7)	0.180
Positive	15	4 (26.7)	7 (46.7)	2 (13.3)	2 (13.3)	
Ki-67						
≤ 14%	29	13 (44.8)	4 (13.8)	4 (13.8)	8 (27.6)	0.012*
> 14%	31	4 (12.9)	11 (35.5)	10 (32.3)	6 (19.3)	
Pairwise comparison: AB: 0.012*, AC:0.012*, AD: 0.441, BC: 1, BD: 0.139, CD: 0.252						
Molecular subtypes						
Luminal A	27	13 (48.2)	2 (7.4)	4 (14.8)	8 (29.6)	<0.001*
Luminal B	24	4 (16.7)	12 (50)	6 (25)	2 (8.3)	
Triple negative	9	0 (0)	1 (11.2)	4 (44.4)	4 (44.4)	
Pairwise comparison: AB: 0.002*, AC: 0.011*, AD: 0.060, BC: 0.108, BD: 0.002*, CD:0.189						

*ER* estrogen receptor, *PR* progesterone receptor, *TAMs* tumor-associated macrophage, *TN* tumor nest, *TS* tumor stroma

*Statistically significant

## Discussion

Breast cancer is the most frequent malignancy among females worldwide. Traditional therapies against breast cancer have been designed to attack tumor cells themselves. Recently, studies have focused on targeting tumor microenvironment in order to reduce treatment resistance and improve patients outcomes [14].

Tumor-associated macrophages are crucial regulators of cancer cells and microenvironment. They modulate tumorigenesis and adjust the response to therapy. Several studies have reported that TAMs are related to poor prognosis in different tumors as hepatocellular carcinoma, gastric cancer, and lung cancer [15]. Several markers are used to label macrophages. CD68 identifies both tumoricidal M1 and tumor-promoting M2 macrophages, whereas CD163 is expressed principally by M2 macrophages [16].

Studies investigated TAMs infiltration within BC were remarkably variable. They used different markers and methods to assess macrophages. Most of them used CD68 alone to assess macrophages [13, 17], while others combined both CD68 and CD163 [18, 19]. Some studies assessed TAMs in different localization (stroma and nest) [13, 17], while others neglected the location of TAMs and counted total TAMs within the tumor [20, 21].

This study aimed to evaluate CD163 expression compared to the widely used CD68 pan-macrophage marker in breast invasive ductal carcinoma. In addition, it focused on assessing the significance of TAMs localization in relation to clinicopathologic parameters.

As regards TAMs infiltration within TN, the present work revealed that high density of CD163-positive TAMs within TN was significantly associated with high tumor grade and increased Ki-67 proliferation index. On the other hand, there was lack of significant associations between high density of CD68-positive TAMs infiltration and all included clinicopathologic parameters.

Gwak et al. and Jeong et al. reported that a high density of TAMs was related to high tumor grade and higher Ki-67 expression in both locations (tumor nest and tumor stroma) [13, 18]. Similarly, studies by Ni et al. and Sousa et al. revealed significant relations between high infiltration of both CD68-positive and CD163-positive TAMs, without addressing specific location, and high histologic grade and increased Ki-67 proliferation index [20, 21].

On the contrary, Ch'ng et al. and Yang and his colleagues demonstrated that increased TAMs in the stroma, not within tumor nest, were correlated with higher tumor grade [17, 22]. However, Yuan et al. noticed a lack of significant association between CD68-positive TAMs density and tumor grade [23].

It was proposed that high-grade tumors may elaborate higher levels of cytokines that recruit and modulate macrophages as monocyte colony-stimulating factors, interleukin-10, and/or transforming growth factor-β resulting in increased density of CD163-positive TAMs within high-grade tumors [21]. Moreover, TAMs may secrete different cytokines and growth factors that provide mitogenic signals to malignant cells [17].

As regards TAMs infiltration within tumor stroma, the present study reported significant associations between stromal TAMs infiltration and poor prognostic parameters. Infiltration of tumor stroma with high density of CD68-positive TAMs was significantly related to large tumor size, vascular invasion, and positive nodal metastasis, whereas, high density of stromal CD163-positive TAMs was significantly associated with large tumor size, positive vascular invasion, the presence of nodal metastasis, advanced stage, and ER-negative expression.

This was in accordance with several studies. Ch’ng et al. reported that only elevated stromal CD68-positive TAMs were associated with poor prognostic features [17]. Also, Medrek et al. showed that high density of only CD68- and CD163-positive stromal TAMs were related to larger tumors and inversely correlated with ER-positive expression and luminal A subtype [19]. Moreover, a study by Gwak et al. showed that high density of CD68-positive TAMs, in both tumor nest and stroma, was associated with aggressive histologic features [13].

Dealing with molecular subtypes, this study observed significant associations between CD163-positive TAMs in both TN and TS and molecular subtypes. Most of triple-negative tumors was associated with high density of CD163 positive TAMs. Whereas, luminal A tumors were accompanied by low levels of CD163 positive TAMs within both TN and TS. Similar results were reported by others [13, 18, 19]. A study by Stossi et al. has demonstrated that conditioned media from macrophages was able to stimulate different pathways inside BC cells that were important for the downregulation of ER expression [24].

Some studies evaluated total TAMs without considering TAMs localization and reported that TAMs were associated with an unfavorable prognosis [20, 23, 25]. The present work demonstrated that stromal TAMs were related to more aggressive behavior than that within the tumor nest. It was suggested that TAMs exert its function through regulating immune response within tumor stroma rather than by direct interaction with tumor cells [22].

The role of TAMs in infiltrating tumor nest differs by tumor type. High density of TAMs within tumor nest correlates with better prognosis in endometrial and gastric carcinomas. However, in malignant melanomas and esophageal cancers, TAMs within tumor nest was found to be associated with poor patient outcome [26]. In the tumor stroma, macrophages are recruited by cytokines produced by malignant cells. Most TAMs in the tumor microenvironment are mainly of M2-like phenotype that secretes high levels of cytokines and thus enhances tumor progression. Moreover, TAMs prevent infiltration and action of CD8+ cytotoxic T lymphocytes in attacking tumor cells [2].

In addition, TAMs express vascular endothelial growth factor (VEGF) and activate angiogenesis, and stimulate tumor cell proliferation and metastasis Moreover, TAMs can generate proteases that degrade extracellular matrix and thus enhance tumor cell invasion [27].

## Conclusion

High density of CD163-positive stromal TAMs is strongly associated with positive vascular invasion, nodal metastasis, and molecular subtypes. Whereas high density of CD68-positive stromal TAMs is related to large tumor size and positive nodal metastasis. TAMs within tumor stroma and tumor nest have different levels of association with poor prognostic parameters. So, it is of great importance to consider the histologic localization of TAMs in addition to the degree of TAMs infiltration.

## Data Availability

The datasets used and/or analyzed during the current study are available from the corresponding author on reasonable request.

## Abbreviations

ANOVA: Analysis of variance
BC: Breast cancer
CI: Confidence interval
DAB: Diaminobenzidine
ER: Estrogen receptor
HRP: Horseradish peroxidase
LN: Lymph node
OR: Odds ratio
PR: Progesterone receptor
TAMs: Tumor-associated macrophages
TME: Tumor microenvironment
TN: Tumor nest
TS: Tumor stroma
VEGF: Vascular endothelial growth factor
WHO: World Health Organization
